# Supplementary material for: Prognostic Value of PD-L1, PD-1 and CD8A in Canine Diffuse Large B-Cell Lymphoma Detected by RNAscope
Source: Vet Sci. 2021 Jun 29;8(7):120. doi: 10.3390/vetsci8070120 (PMC8310184; doi:10.3390/vetsci8070120)
Supplement: Supplementary file 1 [file vetsci-08-00120-s001.zip › Tables S2 and S3.pdf]

Table S2. Median TTP and survival analysis

	TTP (days) (median; range)	p-value			Hazard ratio (95% CI)
		Log-rank test	Cox univariate	Cox multivariate	
Breed		0.971	0.971		
Pure	171; 1-1403				0.984 (0.420-2.307)
Mixed	69; 35-588				ref
Sex		**0.005**	**0.025**	0.088	
Female	95; 35-1403				0.546 (0.250-1.195)
Spayed female	179; 34-325				0.761 (0.403-1.437)
Male	202; 1-988				0.501 (0.268-0.935)
Neutered male	24; 1-37				ref
Age		0.367	0.371		
<7 years	98; 1-1403				1.396 (0.672-2.901)
≥7 years	171; 24-988				Ref
Weight		0.715	0.717		
<10kg	63; 1-455				Ref
≥10kg	171; 1-1403				0.920 (0.585-1.447)
Stage		0.661	0.667		
III	78; 78-202				1.384 (0.314-6.094)
IV	60; 24-493				1.422 (0.636-3.182)
V	171; 1-1403				ref
Sustage		0.090	0.099	**0.004**	
a	180; 1-1403				0.703 (0.462-1.069)
b	69; 1-250				ref
Bone marrow		0.679	0.681		
Infiltrated	69; 1-588				Ref
Not infiltrated	171; 1-1403				0.921 (0.622-1.363)
Blood infiltration (%)			0.823		0.997 (0.967-1.027)
Bone marrow infiltration (%)			0.202	0.221	1.020 (0.989-1.052)
CD8+ cells (flow cytometry, %)			0.579		1.028 (0.931-1.135)
LDH activity		0.939	0.939		
Normal	98; 1-988				Ref
Increased	179; 1-1403				1.014 (0.710-1.448)
Pretreatment with steroids		0.306	0.313		
Yes	98; 1-325				Ref
No	171; 1-1403				0.813 (0.543-1.216)
Treatment		**0.001**	**0.002**	**0.001**	
Chemo	34; 1-455				1.848 (1.254-2.723)
Chemoimmuno	202; 35-1403				Ref
Immunosignature		**0.024**	**0.029**	0.905	
Cold	228; 32-988				0.662 (0.457-0.958)
Hot	60; 1-1403				Ref
PD-L1 (RNAseq)			0.961		1.007 (0.749-1.355)
PD1 (RNAseq)			**0.001**	**0.005**	1.381 (1.138-1.676)
CD8a (RNAseq)			0.082	0.163	1.197 (0.977-1.467)
PD-L1 (RNAcope)		**<0.001**	**<0.001**	**0.002**	

0	179				0.389 (0.044-3.418)
1	273; 180-988				0.058 (0.013-0.250)
2	202; 98-1403				0.059 (0.011-0.306)
3	35; 1-95				1.879 (0.593-5.952)
4	36; 1-196				Ref
PD1 (RNAscope)		**0.010**	**0.019**	0.239	
0	179; 20-493				0.306 (0.097-0.964)
1	288; 35-1403				0.165 (0.051-0.534)
2	69; 1-455				0.555 (0.190-1.618)
3	37; 1-196				Ref
CD8a (RNAscope)		0.182	0.227	0.138	
0	250; 250-273				0.223 (0.028-1.761)
1	98; 1-1403				0.199 (0.036-1.095)
2	196; 32-988				0.271 (0.054-1.372)
3	35; 1-455				0.496 (0.100-2.464)
4	36; 36-60				Ref
Ki67 (%)			**<0.001**	0.877	1.037 (1.020-1.054)
TILs		0.597	0.618		
5	250; 1-1403				0.721 (0.088-5.930)
15	196; 20-493				1.113 (0.128-9.671)
25	95; 1-988				1.326 (0.166-10.609)
40	36; 24-455				1.608 (0.193-13.415)
50	202				Ref

Table S3. Median LSS and survival analysis

	LSS (days) (median; range)	p-value			Hazard ratio (95% CI)
		Log-rank test	Cox univariate	Cox multivariate	
Breed		1.000	1.000		
Pure	237; 22-1403				1.000 (0.421-2.377)
Mixed	82; 50-989				Ref
Sex		0.066	0.096	0.058	
Female	188; 50-1403				0.211 (0.047-0.949)
Spayed female	288; 41-645				0.390 (0.104-1.457)
Male	544; 23-1153				0.196 (0.050-0.768)
Neutered male	28; 22-267				Ref
Age		0.577	0.578		
<7 years	237; 22-1403				Ref
≥7 years	228; 28-1153				1.234 (0.589-2.584)
Weight		0.760	0.760		
<10kg	82; 23-989				Ref
≥10kg	237; 22-1403				0.932 (0.593-1.464)
Stage		0.451	0.458		
III	87; 87-388				1.475 (0.331-6.565)
IV	267; 28-585				1.699 (0.728-3.966)
V	228; 22-1403				Ref
Sustage		0.313	0.317		
a	278; 22-1403				0.806 (0.529-1.299)
b	188; 23-669				Ref

Bone marrow		0.812	0.812		
Infiltrated	71; 22-767				Ref
Not infiltrated	237; 23-1403				0.951 (0.629-1.437)
Blood infiltration (%)			0.617		0.993 (0.964-1.022)
Bone marrow infiltration (%)			0.218	0.077	1.020 (0.988-1.054)
CD8+ cells (flow cytometry, %)			0.437		1.039 (0.943-1.144)
LDH activity		0.940	0.940		
Normal	178; 23-1153				Ref
Increased	267; 22-1403				1.014 (0.705-1.460)
Pretreatment with steroids		0.350	0.353		
Yes	178; 23-669				Ref
No	267; 22-1403				0.827 (0.553-1.235)
Treatment		**0.002**	**0.004**	**0.022**	
Chemo	60; 22-989				1.776 (1.207-2.613)
Chemoimmuno	330; 50-1403				Ref
Immunosignature		**0.010**	**0.012**	0.710	
Cold	544; 60-1153				0.615 (0.420-0.898)
Hot	87; 22-1403				Ref
PD-L1 (RNAseq)			0.789		1.044 (0.762-1.430)
PD1 (RNAseq)			**0.001**	0.182	1.356 (1.134-1.623)
CD8a (RNAseq)			**0.035**	0.135	1.241 (1.015-1.517)
PD-L1 (RNAscope)		**<0.001**	**0.002**	**0.002**	
0	228				0.380 (0.044-3.270)
1	544; 302-1153				0.034 (0.006-0.191)
2	645; 178-1403				0.027 (0.004-0.195)
3	71; 22-278				0.668 (0.235-1.901)
4	50; 23-237				Ref
PD1 (RNAscope)		**0.026**	**0.040**	0.076	
0	228; 35-669				0.340 (0.108-1.071)
1	356; 63-1403				0.191 (0.061-0.600)
2	71; 22-989				0.459 (0.149-1.411)
3	82; 23-267				Ref
CD8a (RNAscope)		0.235	0.283	0.128	
0	302; 302-669				0.199 (0.026-1.549)
1	228; 22-1403				0.220 (0.041-1.173)
2	330; 60-1153				0.240 (0.048-1.210)
3	82; 23-989				0.432 (0.088-2.128)
4	50; 50-90				Ref
Ki67 (%)			**<0.001**	0.102	1.047 (1.027-1.067)
TILs		0.670	0.682		
5	302; 22-1403				0.838 (0.102-6.878)
15	237; 35-585				1.645 (0.190-14.219)
25	188; 23-1153				1.408 (0.174-11.430)
40	94; 28-989				1.666 (0.200-13.865)
50	388				Ref
